# Ly6/uPAR Protein from *Asterias rubens* Starfish Stimulates Migration and Invasion of Human Epithelial and Immune Cells

**DOI:** 10.3390/md24010003

**Published:** 2025-12-19

**Authors:** Ekaterina N. Lyukmanova, Tamara Y. Gornostaeva, Sergey V. Shabelnikov, Zakhar O. Shenkarev, Mikhail P. Kirpichnikov, Alexander S. Paramonov, Maxim L. Bychkov

**Affiliations:** 1 Faculty of Biology, Shenzhen MSU-BIT University, Shenzhen 518172, China; kirpichnikov@inbox.ru; 2 Shemyakin-Ovchinnikov Institute of Bioorganic Chemistry, Russian Academy of Sciences, Moscow 117997, Russia; gornostaevatamara@gmail.com (T.Y.G.); zakhar-shenkarev@yandex.ru (Z.O.S.);; 3 Moscow Center for Advanced Studies, Moscow 123592, Russia; 4 Interdisciplinary Scientific and Educational School of Moscow University “Molecular Technologies of the Living Systems and Synthetic Biology”, Faculty of Biology, Lomonosov Moscow State University, Moscow 119234, Russia; 5 Institute of Cytology, Russian Academy of Sciences, Tikhoretsky Prospect 4, St. Petersburg 194064, Russia; buddasvami@gmail.com

**Keywords:** three-finger protein, nicotinic acetylcholine receptor, integrin, E/N cadherin switch, E-selectin, wound healing, Lystar5

## Abstract

Recently, we found that Lystar5 protein from coelomic cells of *A. rubens* starfish interacts with nicotinic acetylcholine receptors (nAChRs) and integrin α8-like protein. We hypothesized that Lystar5 mediates detachment of coelomic cells from the matrix and their migration. Skin wound healing in humans is based on keratinocytes migration and is regulated by nAChRs and integrins. Here, we revealed that Lystar5 stimulates migration of human skin HaCaT keratinocytes and peripheral blood monocytes. Using ELISA, we found that Lystar5 binds to the membrane fraction of coelomic cells with its loops I and II, which form an active site of Lystar5 and resemble its pro-migratory activity. In keratinocytes and monocytes, Lystar5 and the peptides mimicking its loops I and II bound with α3, α4, and β2 nAChR and α5, αV, and β1 integrin subunits, which form molecular complexes. In keratinocytes, Lystar5 and its mimetics promoted short-term E/N cadherin switch and upregulated expression of α5 and αV integrins, EGFR, and ICAM-1. In keratinocytes and monocytes, Lystar5 and its mimetics upregulated E-selectin secretion. The ability of Lystar5 and its mimetics to stimulate skin keratinocyte migration and immune cell infiltration may be considered promising for the development of new wound-healing agents.

## 1. Introduction

Wound healing is a highly organized dynamic process, in which a balance between proliferation and cell migration determines the success of epithelial restoration [1]. Keratinocyte migration is especially necessary to maintain the barrier function of the skin, so this process often is identified as a crucial stage in the wound healing [2,3]. Control of keratinocyte migration is carried out by the various growth factors and signaling pathways [4]. At the same time, in the case of a deep wound, the immune system actively participates in the healing process both on the level of immunomodulatory cytokines and secreted factors [1,4,5] and on the level of infiltration of immune cells that ensure control of inflammation and barrier restoration [6,7].

Nicotinic acetylcholine receptors (nAChRs) are ligand-gated pentameric ion channels, which are expressed not only in the nervous system, but also in non-neuronal cells and tissues, and can be activated by acetylcholine or nicotine [8,9]. Non-neuronal nAChRs are involved in the regulation of the immune system [10] and the growth, migration, and differentiation of non-neuronal cells [8,9]. nAChRs containing the α3, α4, α5, α7, α9, β2, and β4 subunits regulate the differentiation, growth, and migration of keratinocytes in the wound healing [11,12,13,14,15,16,17], while inflammation during wound healing is controlled by modulation of α7-AChRs in skin cells and monocytes infiltrating the wound [11,18,19,20,21,22]. Thus, the role of nAChRs in wound healing can include both the regulation of the migration of keratinocytes [11,12,14,15,16] and the control of inflammation [19,20,22]. Besides nAChRs, the wound repair is mediated by fibronectin receptors such as α5 and αV integrins, which are upregulated in the damaged epithelium [23] and regulate both keratinocyte migration and leukocyte infiltration during wound re-epithelization [24]. Thus, both nAChRs and integrins are directly involved in wound healing and their simultaneous targeting may become a promising strategy for wound therapy, including non-healing and deep wounds.

Earlier, we discovered and, for the first time, characterized the three-finger protein Lystar5 from the starfish *Asterias rubens* [25,26]. The Three-Finger Protein family (also known as Ly6/uPAR family) includes relatively small (usually 60–90 residues), cysteine-rich (4–6 disulfides) proteins with a characteristic (“three-finger”) fold consisting of a compact β-structural core with three protruding loops (“fingers”). These loop regions are suggested to be responsible for interaction with various targets of Ly6/uPAR proteins [27]. Ly6/uPAR proteins were found in many organisms [27], and the most famous representatives of them are snake neurotoxins that target many essential receptors of mammals, including nAChRs [28]. Lystar5 demonstrates high sequence similarity with human three-finger neuromodulator Lynx2 (Lypd1), which is related to anxiety [29,30]. The study of the secondary structure of recombinant Lystar5 (91 residues, Mw 10.3 kDa) by NMR-spectroscopy confirmed the three-finger organization of the protein [25]. The function of Lystar5 in the starfish organism is not clear yet. It was shown that Lystar5 inhibits a highly sensitive isoform of α4β2-nAChRs [25], is presented on the surface of the coelomocytes and coelomic epithelium, and interacts with the integrin α8-like protein [26]. Lystar5 was hypothesized to mediate the detachment of the coelomic epithelial cells during their transformation into the coelomocytes. Coelomic epithelial cells serve as a barrier and subsequently participate in the immune response after their detachment [31,32]. Since Lystar5 is expressed both in the coelomic epithelial cells before their detachment and in the coelomocytes, we hypothesized that this protein may regulate epithelial cell motility and immune processes in starfish. On the other hand, the integrin α8-like protein shares a high degree of similarity with human α5 and αV integrins (Figure S1), which are involved in wound healing. However, the potential of Lystar5 application as a wound-healing or immunomodulatory agent has not been investigated yet.

In this study, we demonstrated for the first time that Lystar5 can stimulate the migration of human keratinocytes and monocytes, identified the active site of Lystar5, and investigated the wound-healing potential of the peptides mimicking its active site. The results of our study have great implications for the development of new approaches for wound treatment.

## 2. Results

### 2.1. Lystar5 Stimulates Migration of Skin Keratinocytes

**Figure 1:**
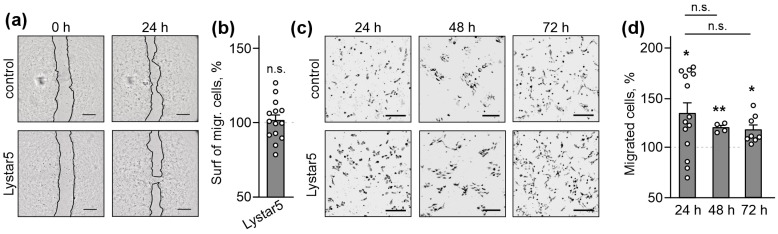
Influence of Lystar5 on the collective and individual migration of human HaCaT skin keratinocytes. HaCaT keratinocytes were incubated with 1 µM of Lystar5 for 24, 48 or 72 h. (**a**) Representative pictures of the scratch assay for collective migration. Scale = 500 µm. (**b**) Quantification of the surface occupied by the migrated cells. Data are presented as the normalized surface of the migrated cells (% of the control (untreated cells), 100%, dashed line) ± SEM (*n* = 14). n.s.—no significant difference. (**c**) Representative pictures of the migrated cells through the chamber pores. Scale = 200 µm. (**d**) Quantification of the migrated cells. Data are presented as the normalized number of the migrated cells (% of the control (untreated cells), 100%, dashed line) ± SEM (*n* = 4–14). * (*p* < 0.05) and ** (*p* < 0.01) indicate the significant difference from the control according to one sample two-tailed *t*-test followed by Holm–Sidak’s *post hoc* test.

Lystar5 was found to interact with the integrin α8-like protein [26], while integrins can be involved in the regulation of cell migration [33]. Thus, we suggested that Lystar5 could affect cell migration. To prove this hypothesis, we tested whether incubation with Lystar5 may influence the collective or individual migration of human skin HaCaT keratinocytes. A scratch wound-healing assay revealed that Lystar5 did not affect the collective migration of HaCaT keratinocytes upon 24 h incubation (Figure 1a,b). Notably, we did not evaluate skin keratinocyte migration in the scratch assay upon longer incubation with Lystar5 due to cell overgrowth and death (see the Methods Section for more details about the study design).

To study Lystar5’s influence on the individual migration of HaCaT keratinocytes, we performed the assay with 8 µm pore migration chambers. It was revealed that 24 h of incubation with Lystar5 significantly stimulates the individual migration of HaCaT keratinocytes through the chamber pores (Figure 1c,d), and this effect lasts at least 72 h (Figure 1c,d). Thus, Lystar5 can indeed stimulate the migration of human skin keratinocytes.

### 2.2. Loops I and II of Lystar5 Form Its Active Site

Recently, we have showed that the recombinant analogue of Lystar5 targets the proteins from the membrane fraction of coelomocytes and coelomic epithelium [26]. Here, we studied the active site of Lystar5. Lystar5 belongs to the Ly6/uPAR protein family, for which the loop regions usually are considered functional epitopes [27]. To determine the active site of Lystar5, we used the previously developed strategy based on the study of the activity of the peptides mimicking the loops of a Ly6/uPAR protein [34,35,36].

**Figure 2:**
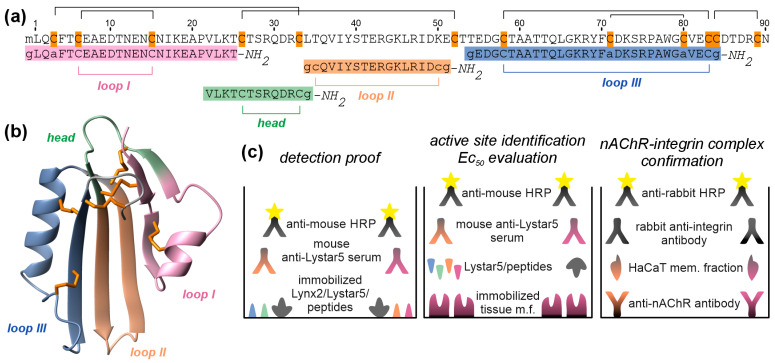
Design of the peptides mimicking the loop and “head” regions of Lystar5 and ELISA experiments. (**a**) Amino acid sequence alignment of Lystar5 and the peptides: loop I (rose), loop II (orange), loop III (blue), and head (green). Cys residues are shown in gold. Disulfide bonds are shown in brackets. Sequences of the peptides are highlighted by colors corresponding to the Lystar5 loops. The amino acids changed to increase the peptides’ stability are designated by lowercase letters. (**b**) Spatial structure of Lystar5 predicted from the sequence using ESMFold [37] with regions corresponding to the synthesized peptides colored as shown in panel (**a**,**c**). Design of ELISA experiments for proof of detection of Lystar5 and the peptides by the anti-Lystar5 serum of immunized mice (**left**), identification of the active site of Lystar5, and evaluation of the interaction of Lystar5 and the peptides using the immobilized membrane fraction of different *A. rubens* tissues (**center**), and study of complex formation between nAChR and integrin subunits in HaCAT keratinocytes (**right**). To prove recognition of the anti-Lystar5 serum, Lystar5 and the peptides mimicking its loops and “head” were immobilized to ELISA wells. Recombinant analogue of Lynx2 was used as a negative control. For active site identification and EC_50_ estimation, membrane fraction of the coelomic cells, coelomic epithelium, and arm tip of *A. rubens* was immobilized to wells of ELISA plate and incubated with different amounts of Lystar5 and peptides. Bound compounds were detected by the anti-Lystar5 serum of immunized mouse. To prove nAChR/integrin complex formation, the antibodies to nAChR subunits were immobilized in the wells of ELISA plate, which was then incubated with the membrane fraction of HaCaT cells, and then the binding of different integrin subunits was detected with anti-integrin antibodies (see the Methods Section for further details).

**Figure 3:**
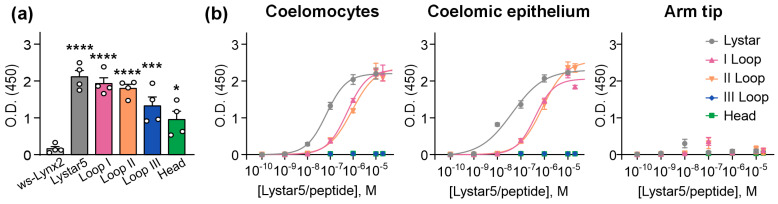
Identification of the Lystar5 active site by ELISA. (**a**) Detection of immobilized Lystar5, peptides, and ws-Lynx2 by mouse anti-Lystar5 serum. Data are presented as background-subtracted O.D. ± SEM (*n* = 4). * (*p* < 0.05), *** (*p* < 0.001), and **** (*p* < 0.0001) indicate the difference from the ws-Lynx2 group according to one-way ANOVA followed by Dunnet’s *post hoc* test. (**b**) Interaction of Lystar5 and the peptides with the immobilized membrane fraction of different tissues of *A. rubens*. Parameters describing the fit of dose–response curves for interaction of Lystar5 and loops I and II with the membrane fraction of coelomic cells are given in Table 1. Data are presented as background-subtracted O.D. ± SEM (*n* = 4).

We designed the peptides mimicking the Lystar5 loops and “head” regions (Figure 2a,b) and compared their ability to bind with the membrane fraction of different *A. rubens* tissues using ELISA (Figure 2c and Figure 3). Amino acid sequences of the peptides and Lystar5 are shown in Figure 2a. Notably, the *C*-termini of the peptides were amidated, and the Cys residues in the peptides mimicking loops I and III which did not participate in the disulfide bond formation were replaced by the Ala residues.

For detection of Lystar5 and the peptides mimicking its loops and “head” bound to the immobilized membrane fraction of the coelomic cells, coelomic epithelium, and arm tip of *A. rubens* by ELISA, the serum of the mice immunized by recombinant Lystar5 was used. Although all peptides were detected by the anti-Lystar5 serum (Figure 3a), only Lystar5 and the peptides mimicking loops I and II bonded to the membrane fraction of the coelomic cells and coelomic epithelium (Figure 3b). Notably, the specificity of the anti-Lystar5 serum was confirmed by the usage of a recombinant analogue of human Ly6/uPAR protein ws-Lynx2 [38], which was not recognized by the anti-Lystar5 serum (Figure 3a).

Similarly to Lystar5, the peptides mimicking loops I and II demonstrated the dose–response curves of the interaction with the membrane fraction of the coelomic cells (Figure 3b), although with the higher EC_50_ values in comparison with the EC_50_ value of Lystar5 (Table 1). As in [26], neither Lystar5 nor its loops or “head” bound to the immobilized membrane fraction of the arm tip, pointing to the specificity of the interaction of Lystar5 and the peptides with the coelomic cells. Thus, loops I and II were identified as the active site of Lystar5, which interact with its targets in the coelomocytes and coelomic epithelium of *A. rubens*.

### 2.3. Peptides Mimicking Loops I and II Stimulate Migration of Skin Keratinocytes

**Figure 4:**
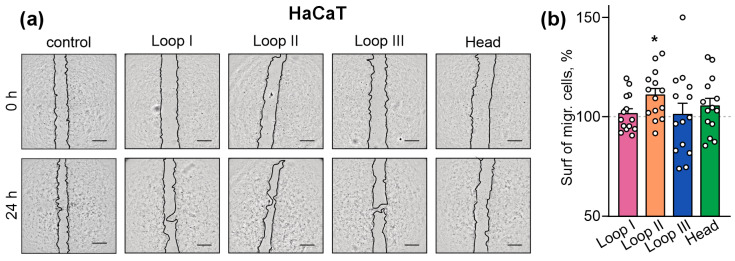
Influence of the peptides mimicking the loops and “head” of Lystar5 on the collective migration of HaCaT skin keratinocytes. HaCaT keratinocytes were incubated with 10 µM of the peptides for 24 h. Representative pictures of the scratch assay (**a**) and the quantification of the surface occupied by the migrated keratinocytes (**b**) are shown. Data are presented as normalized square occupied by the migrated cells (% of the control (untreated cells), 100%, dashed line) ± SEM (*n* = 14). Scale = 500 µm. * (*p* < 0.05) indicates the significant difference from the control according to one sample two-tailed t-test followed by Holm–Sidak’s *post hoc* test.

To prove that the peptides mimicking the active site of Lystar5 have the ability to mimic its biological activity, we tested the influence of the peptides mimicking the Lystar5 loops and “head” on the HaCaT keratinocytes migration. The scratch wound-healing assay revealed that the peptides mimicking loops I and III, as well as “head,” did not affect the collective migration of HaCaT keratinocytes upon 24 h of incubation (Figure 4a,b). However, the peptide mimicking the loop II of Lystar5 significantly increased the wound closure in the HaCaT scratch (Figure 4a,b).

**Figure 5:**
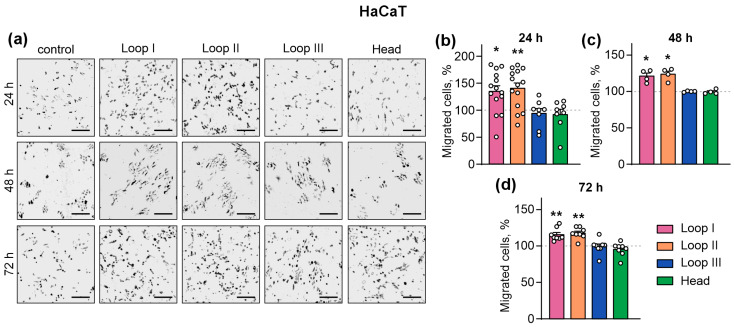
Influence of the peptides mimicking its loops and “head” on migration of individual HaCaT skin keratinocytes in the test with 8-µm pore chambers. HaCaT keratinocytes were incubated with 10 µM of the peptides for 24, 48 or 72 h. The representative pictures of the migrated cells (**a**) and quantification of the migrated cells (**b**–**d**) are shown. Scale = 200 µm. Data are presented as normalized number of the migrated cells (% of the control (untreated cells), 100%, dashed line) ± SEM (*n* = 4–14). * (*p* < 0.05) and ** (*p* < 0.01) indicate the significant difference from the control according to one sample two-tailed *t*-test followed by Holm–Sidak’s *post hoc* test.

Incubation during 24 h with the peptides mimicking loops I and II of Lystar5, but not loop III or the “head,” significantly stimulated the individual migration of HaCaT keratinocytes (Figure 5a,b), and, similarly to Lystar5, this effect lasted for 48 and 72 h (Figure 5a,c). Thus, indeed, the peptides mimicking the active site of Lystar5 can stimulate collective and individual migration of skin keratinocytes, and loop I increases the individual migration of HaCaT keratinocytes, while loop II of the protein stimulates both individual and collective keratinocyte migration.

### 2.4. Lystar5 and the Peptide Mimicking Loop II Stimulate the Infiltration of Peripheral Blood Monocytes

**Figure 6:**
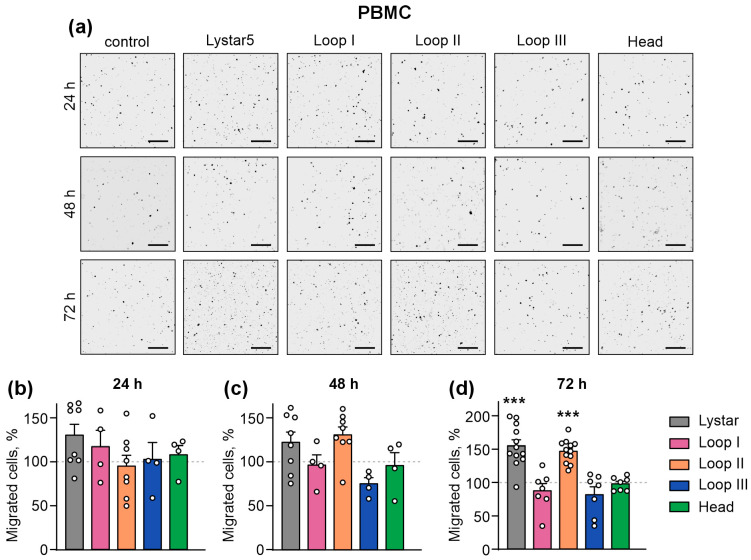
Influence of Lystar5 and the peptides mimicking its loops and “head” on the migration of PBMCs in the test with 8 µm pore chambers. PBMCs were incubated with 1 µM of Lystar5 or 10 µM of the peptides for 24, 48, or 72 h. The representative pictures of migrated cells (**a**) and quantification of the migrated cells (**b**–**d**) are shown. Scale = 200 µm. Data are presented as the normalized number of the migrated cells (% of the control (untreated cells), 100%, dashed line) ± SEM (*n* = 4–12). *** (*p* < 0.001) indicates the significant difference from the control according to one sample two-tailed *t*-test followed by Holm–Sidak *post hoc* test.

There are several human Ly6/uPAR proteins regulating the immune cells [39,40]. Here, we elucidated whether Lystar5 or its peptide mimetics can affect the migration of peripheral blood monocytes (PBMCs), which represent a mixture of T and B lymphocytes, monocytes, and dendritic cells circulating in the peripheral blood [41]. All these cells orchestrate inflammation and support keratinocytes’ proliferation, migration, and ECM remodeling in the wound site [42,43]. To model the PBMCs’ infiltration into the wound site, we performed a migration chamber assay. No influence of Lystar5 and the peptides on PBMC migration was revealed following 24 or 48 h of incubation (Figure 6a,b), while both Lystar5 and loop II significantly increased the number of migrated PBMCs upon 72 h of incubation (Figure 6a,c).

### 2.5. Lystar5 and the Peptides Mimicking Loops I and II Target nAChRs and Integrins in Skin Keratinocytes and PBMCs

**Figure 7:**
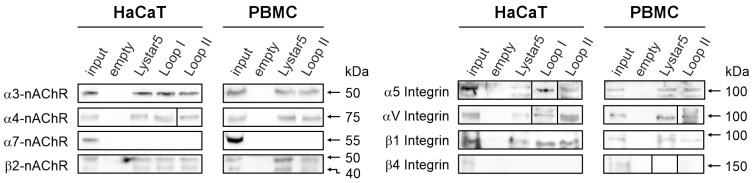
Analysis of the targets of Lystar5 and the peptides mimicking loops I and II in the membrane fraction of HaCaT keratinocytes and PBMCs. Lystar5 or the peptides were coupled with NHS Flexibind magnetic beads, incubated with the membrane fraction of HaCaT keratinocytes or PBMCs, and bound targets were analyzed by Western blotting (*n* = 3 independent portions of cells). Whole Western blotting membranes are shown in Figures S2–S5. “Empty” samples correspond to the proteins extracted from the membrane fraction by the magnetic beads blocked by 500 mM ethanolamine without any compounds coupled. Beads coupled with BSA served as a control of extraction specificity (Figure S6).

Previously, we have shown that Lystar5 inhibits the highly sensitive isoform of α4β2-nAChR expressed in *Xenopus* oocytes [25] and extracts from the membrane fraction of *A. rubens* coelomocytes the integrin α-8-like protein (XP_033639394.1) [26]. According to BLASTP analysis, the integrin α-8-like protein of *A. rubens* demonstrates relatively high sequence identity (31%) and similarity (50% and 49%, respectively) with α-subunits of two human integrins: α5 (UniProt accession code P08648) and αV (UniProt accession code P06756) (Figure S1). We proposed that the interaction with the integrins can mediate the effects of Lystar5 and its peptide mimetics in skin keratinocytes and PBMCs. We performed an affinity extraction of the targets of Lystar5 and the peptides mimicking its loops I and II from the membrane fraction of HaCaT keratinocytes and PBMCs. Western blotting analysis revealed that Lystar5 and loops I and II bind α3, α4, and β2, but not α7 nAChR subunits in HaCaT keratinocytes (Figure 7). Additionally, Lystar5 and loops I and II extracted α5, αV, and β1, but not β4 integrin subunits (Figure 7). A similar profile of the extracted targets was revealed in PBMCs, but only for Lystar5 and the peptide mimicking loop II (Figure 7).

### 2.6. nAChRs Form Complexes with Integrins in HaCaT Keratinocytes

**Figure 8:**
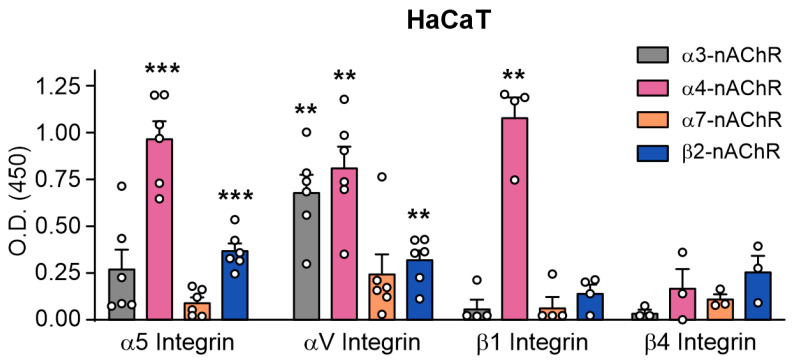
Complex formation between nAChRs and integrins in HaCaT keratinocytes. Antibodies to nAChR subunits were immobilized in the ELISA plate wells, then the plate was incubated with the membrane fraction of HaCaT keratinocytes and different integrin subunits bound to the wells via interaction with nAChR subunits were detected by specific antibodies (see the Methods Section for negative controls details). Data are presented as background-subtracted O.D. ± SEM (*n* = 3–6). ** (*p* < 0.01) and *** (*p* < 0.001) indicate the difference from the background according to one sample two-tailed *t*-test followed by Holm–Sidak’s *post hoc* test.

Lystar5 targeting of both nAChR and integrin subunits in the keratinocytes and PBMCs (Figure 7) suggested that nAChRs and integrins can form the complexes in these cells. To study this, we designed an ELISA, in which the membrane fraction of HaCaT cells was incubated in wells with immobilized antibodies to α3, α4, α7, and β2 nAChR subunits. Then, the α5, αV, β1, and β4 integrin subunits bound to the wells via possible interaction with nAChRs were detected by respective anti-integrin antibodies. We found that nAChRs containing the α3 subunit formed the complex only with αV integrin, while nAChRs containing the α4 subunit bound to α5, αV, and β1 integrins (Figure 8). Also, nAChRs containing the β2 subunit bound α5 and αV integrin subunits. The binding of β4 integrin with all nAChR subunits studied was insignificant (Figure 8). Thus, our results confirmed the formation of complexes between nAChRs and integrins in HaCaT keratinocytes. It is possible that Lystar5 and its mimetics interact not solely with nAChRs and integrins, but with a network formed by these signaling receptors.

### 2.7. Lystar5 and the Peptides Mimicking Loops I and II Affect Migration-Related Factors in Skin Keratinocytes and PBMCs

Wound healing is mediated by activation of surface receptors and secretion of different paracrine factors by keratinocytes and immune cells [1,3]. We used flow cytometry to analyze the influence of the incubation of HaCaT keratinocytes and PBMCs with Lystar5 and the peptides mimicking loops I and II on the cell surface expression of adhesion molecules (E- and N- cadherins, α5 and αV integrins, ICAM-1, and VCAM-1) and pro-migratory receptor tyrosine-kinases EGFR and VEGFR2.

**Figure 9:**
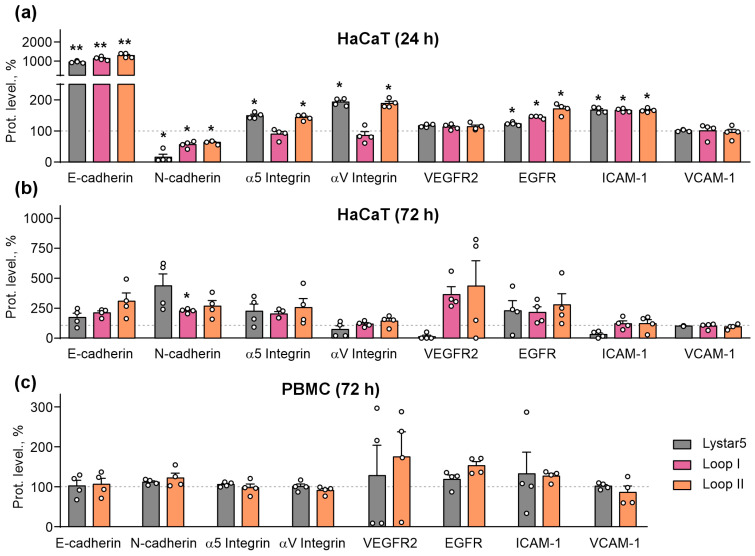
Influence of Lystar5 and the peptides mimicking loops I and II on the cell surface expression of migration-related adhesion molecules and receptors in HaCaT keratinocytes (**a**,**b**) and PBMCs (**c**). HaCaT keratinocytes were incubated with 1 µM of Lystar5 or 10 µM of the peptides for 24 h (**a**) or 72 h (**b**). PBMCs were incubated with 1 µM of Lystar5 or 10 µM of the peptide mimicking loop II for 72 h (**c**). Cell surface expression of migration-related factors was assayed by flow cytometry. Representative cell distribution histograms showing the expression of assayed molecules are in Figure S7. Data present the normalized MFI (% of the control (untreated cells), 100%, dashed line) ± SEM (*n* = 4). * (*p* < 0.05) and ** (*p* < 0.01) indicate the significant difference from the control according to one sample two-tailed *t*-test followed by Holm–Sidak’s *post hoc* test.

It was found that 24 h of incubation with Lystar5 and the peptides mimicking loops I and II upregulated the expression of E-cadherin, EGFR, and ICAM-1 in HaCaT keratinocytes (Figure 9a). At the same time, all compounds downregulated the surface expression of anti-migratory factor N-cadherin (Figure 9a). Simultaneous upregulation of pro-migratory E-cadherin and downregulation of anti-migratory N-cadherin points to the E/N cadherin switch, which is characteristic for the migrating cells [44]. Moreover, Lystar5 and the peptide mimicking loop II upregulated the surface expression of α5 and αV integrins (Figure 9a). All these effects were diminished upon 72 h incubation of HaCaT cells with the compounds (Figure 9b). In contrast with the keratinocytes, no influence of PBMCs incubation with Lystar5 and the peptide mimicking loop II on the cell surface expression of the studied adhesion molecules was revealed (Figure 9c).

**Figure 10:**
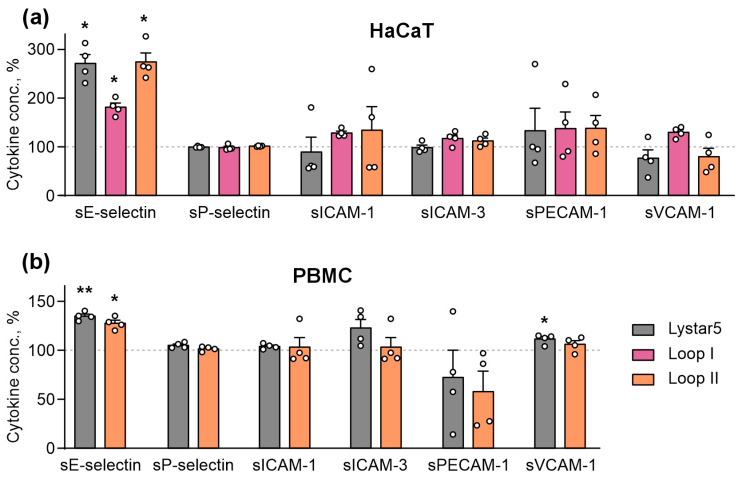
Influence of Lystar5 and the peptides mimicking loops I and II on the secretion of migration-related adhesion and inflammatory factors in HaCaT keratinocytes (**a**) and PBMCs (**b**). HaCaT keratinocytes were incubated with 1 µM of Lystar5 or 10 µM of the peptides for 24 h. PBMCs were incubated with 1 µM of Lystar5 or 10 µM of the peptide mimicking the loop II for 72 h. Secretion of soluble factors was assayed using the Flow cytomix 6-plex adhesion kit. Data present normalized MFI (% of the control (untreated cells), 100%, dashed line) ± SEM (*n* = 4). * (*p* < 0.05) and ** (*p* < 0.01) indicate the significant difference from the control according to one sample two-tailed *t*-test followed by Holm–Sidak’s *post hoc* test.

Next, we analyzed whether Lystar5 or its mimetics modulate the secretion of adhesion factors by HaCaT cells and PBMCs using the Flow cytomix immunoassay for the detection of soluble adhesion and inflammation-related factors: sE-selectin, sP-selectin, sICAM-1, sICAM-3, sPECAM-1, and sVCAM-1. Index “s” points to the secreted/soluble form of the studied molecules. Immunoassay showed that 24 h incubation of HaCaT cells with Lystar5 and the peptides mimicking loops I and II upregulated the secretion of sE-selectin, while no influence of the compounds on the secretion of other factors was revealed (Figure 10a). Similarly, 72 h incubation of PBMCs with Lystar5 and the peptide mimicking loop II stimulated the secretion of sE-selectin, while Lystar5 also upregulated the secretion of sVCAM-1 adhesion factor (Figure 10b).

Thus, Lystar5 and the mimetics of its active site promoted a short-term E/N-cadherin switch, upregulated cell surface expression of pro-migratory factors and receptors, and stimulated the secretion of E-selectin in HaCaT keratinocytes, while only upregulation of E-selectin secretion was observed in PBMCs.

## 3. Discussion

Ly6/uPAR proteins from different organisms are currently being considered as prototypes for new drugs. Thus, snake α-cobratoxin from *Naja kaouthia* [45], human-secreted epithelial protein SLURP-1 [46], as well as human protein Lynx1 found in the nervous system, skin, and lungs [47,48] were demonstrated to be effective anti-cancer agents. Lynx1 also ameliorates cognitive dysfunctions related to the cholinergic system decline [49], while human-secreted epithelial protein SLURP-2 enhances wound healing [34,50], snake neurotoxin WTX from *Naja kaouthia* showed an ability to normalize blood pressure [51], and mambalgins from mamba venoms inhibit pain [52].

However, the design of new drugs based on Ly6/uPAR proteins is limited by their complicated spatial structure stabilized by the system of 4–6 disulfide bonds (Figure 2b) and difficulty obtaining their functional analogues in the folded state in large amounts. We suggested a new strategy based on the design of peptide mimetics, which resemble useful properties of Ly6/uPAR proteins, and can be simply produced by chemical synthesis or protein engineering. This strategy includes several steps: (1) studying the biological activity of Ly6/uPAR proteins; (2) the identification of active sites of Ly6/uPAR proteins, usually located in their loops [27]; (3) chemical synthesis or recombinant production of peptides mimicking the active site of Ly6/uPAR proteins; (4) characterization of the biological activity of designed peptides; (5) the choice of the prototype for further drug design. We already approved this strategy for the human proteins Lynx1 [36], SLURP-1 [35,46], and SLURP-2 [34]. In this study, we applied this strategy to Lystar5, -Ly6/uPAR protein from starfish *A. rubens* [25,26], and tested whether Lystar5 or its mimetics can be used as wound-healing agents.

First, we studied the biological activity of Lystar5. Previously, we revealed that Lystar5 interacts with the integrin α8-like protein in *A. rubens* coelomic cells, probably promoting coelomocytes’ detachment from the coelomic epithelium [26]. Integrin α8-like protein is homologous to human α5 and αV integrins (Figure S1), which regulate keratinocyte migration during wound healing [23,24]. On the other hand, *A. rubens* coelomic cells participate in starfish tissue regeneration [31]. Thus, we hypothesized the possible involvement of Lystar5 in regulation of coelomic cell motility, and that this Lystar5 function can be translated into human cells. We tested this hypothesis using human HaCaT keratinocytes and peripheral blood monocytes because coelomic cells simultaneously perform a protective function and provide starfish with immunity [32,53]. Indeed, Lystar5 stimulated the migration of individual HaCaT keratinocytes through the 8 µm pores (Figure 1).

Second, we designed the peptide mimetics of Lystar5 based on the loop regions revealed in the analysis of its secondary structure [38] (Figure 2) and found that the active site of Lystar5, which mediates the binding of the protein to the membrane fraction of coelomic cells of *A. rubens*, is formed by loops I and II (Figure 3). Notably, the EC_50_ values of the binding of the peptides mimicking loops I and II of Lystar5 were ~10–20 times higher than that of full-length Lystar5 (Table 1). This could point to the importance of cooperative interaction of different parts of the Lystar5 molecule with its targets. Similarly to Lystar5, both peptides mimicking loops I and II stimulated the migration of individual skin keratinocytes (Figure 5), while loop II resembled Lystar5’s ability to stimulate the migration of individual PBMCs (Figure 6).

Stimulation of the individual migration of keratinocytes by Lystar5 and its loops points to the action mainly affecting the keratinocytes on the edge of the wound, which must loosen their adhesion to each other and basal membrane to migrate and close the wound [54]. However, the stimulation of wound healing in the scratch assay, which reflects the collective migration observed only upon incubation of the keratinocytes with the peptide mimicking loop II of Lystar5 (Figure 4), probably means that its action is linked to the movement of all (not only marginal) keratinocytes. Thus, we assume that Lystar5 and loop I stimulate the primary stages of wound healing, while loop II also supports the migration of keratinocytes into the depths untouched by the wound. Interestingly, the pro-migratory effect of Lystar5 and its mimetics manifested after 24 h of incubation and lasted at least for 72 h (Figure 1 and Figure 5), pointing to the long-term wound-healing support of these compounds.

Monocyte infiltration is also characteristic of wound repair [54]. Both Lystar5 and loop II stimulated the PBMCs’ migration, although they required a longer incubation time in comparison with HaCaT keratinocytes (72 h vs. 24 h, Figure 1, Figure 5 and Figure 6). We suppose that such a time gap can be explained by the different nature of the cells studied (keratinocytes vs. monocytes). At the same time, the PBMCs’ infiltration upon thermal injury in vivo also required 72 h [55]. We can suggest the following sequential steps of the wound-healing process promoted by Lystar5 and loops I and II: (1) initially, the keratinocytes on the wound edge are stimulated, (2) then keratinocytes migrate and cover up the wound, (3) and finally, the monocytes’ infiltration is mediated to support the inflammation required for the proliferation of the keratinocytes, which migrate to the wound site for wound re-epithelization [56].

An affinity extraction experiment revealed that Lystar5 and the peptides mimicking loops I and II interact with different nAChR subunits and integrins (Figure 7). The ability of nAChRs to directly interact with other signaling molecules and receptors was previously reported for receptor tyrosine kinases EGFR or VEGFR [57], IP3K [58], Janus Kinase 2 [58], G-proteins [59], 14-3-3 protein [60,61], and multiple cytoskeletal proteins [62]. Moreover, the activation of α7-nAChR leads to upregulation of α2 integrin expression in keratinocytes [63]. In line with this, short-term incubation of the HaCaT keratinocytes with Lystar5 or its peptide mimetics, which regulate α4β2-nAChRs [25], results in upregulation of α5 and αV integrins expression in keratinocytes (Figure 9a). At the same time, we also observed short-term upregulation of EGFR expression in keratinocytes in response to the Lystar5/mimetics treatment. During wound healing, EGFR can interact with integrins [23] and stimulate keratinocyte migration [64]. Altogether, we propose that Lystar5 targets the complex functional network formed by at least nAChRs, integrins, and EGFR. Indeed, formation of the complexes between nAChRs and integrins was confirmed by immunoprecipitation (Figure 8).

Additionally, incubation of the HaCaT keratinocytes with Lystar5 and its mimetics resulted in upregulated expression of ICAM-1 (Figure 9a). Notably, in addition to mediation of keratinocyte migration, ICAM-1 also promotes infiltration of immune cells into the wound [65] and participates in regulation of the inflammation [66]. Although, ICAM-1 upregulation in the keratinocytes upon the incubation with Lystar5/mimetics lasted only 24 h (Figure 9a,b), this short-term increase in the expression level can have a significant impact on the modulation of the immune response and internal communication between skin and immune cells.

Wound healing is regulated by many other molecules like cadherins, selectins, adhesion factors, and cytokines [67]. We showed that short-term incubation of the keratinocytes with Lystar5 and its mimetics induces the E/N cadherin switch (Figure 9). The E/N cadherin switch mediates cell detachment from the basal matrix required for the migration of individual cells; however, this process is considered to be pro-oncogenic [68]. The short time of the E/N cadherin switch, which disappeared within 72 h (Figure 9b), points toward the pro-migratory effect of Lystar5/mimetics without stimulation of the keratinocytes for oncotransformation. Interestingly, there is crosstalk between the integrins and cadherins [69]. In particular, N-cadherin negatively regulates α5 integrin and the α5 integrin-mediated ECM remodeling during embryogenesis [70]. Also, β1 integrin induces N-cadherin-mediated clustering of neural crest cells [71]. Thus, the binding of Lystar5 or its mimetics to α5 and β1 integrins (Figure 7) supplemented with downregulation of N-cadherin expression and upregulation of α5 integrin expression (Figure 9a) may provide short-term stimulation of keratinocyte motility and possible ECM remodeling. αV integrin also mediates the migration of mammary epithelial cells [72], so it may be implicated in the short-term E/N switch in the HaCaT keratinocytes, too.

Although E-selectin is not directly involved in keratinocytes’ migration, it is upregulated in wounds [73] and, together with integrins, controls the inflammation and recruitment of immune cells into the wound [74]. Thus, the observed upregulation of secreted E-selectin both in keratinocytes and PBMCs (Figure 10) points to Lystar5 potentially having a role both in epithelial homeostasis and in immune cell recruitment into the wound site.

Only Lystar5 and loop II, but not loop I, caused an increase in α5 and αV expression in the keratinocytes (Figure 9a). Moreover, the migration of PBMCs was also stimulated only by Lystar5 and loop II (Figure 6a,c). This difference may be linked to different implications of the loops of Lystar5 in the interaction and, in turn, regulation of the network formed by the nAChRs/integrins/EGFR/etc., which could be addressed further. Specific changes in the expression level of different cell surface receptors only in the keratinocytes, but not in PBMCs, highlight the different mechanisms underlying the migration of these cells.

## 4. Materials and Methods

### 4.1. Recombinant Protein Production

Lystar5 was obtained by recombinant expression in *E. coli*, as described in [25,26]. Ws-Lynx2 (used as a negative control in the ELISA experiment) was obtained as described in [38,75]. The *C*-amidated forms of the peptides (“loop I”: GLQAFTCEAEDTNENCNIKEAPVLKT; “loop II”: GCQVIYSTERGKLRIDCG) “loop III”: GEDGCTAATTQLGKRYFADKSRPAWGAVECG, and “head”: VLKTCTSRQDRCG, were obtained by solid-phase chemical synthesis in Genscript (Nanjing, China). Sequence alignment of peptides and Lystar5 is shown in Figure 2a,b. The homogeneity and purity (>95%) of peptides were confirmed by HPLC, MALDI-MS, and 1D ^1^H-NMR spectroscopy (Figure S8).

### 4.2. A. rubens Tissue Collection

Adult individuals of *A. rubens* starfish were collected at the Biological Station of the Zoological Institute, Russian Academy of Sciences, on Cape Kartesh (Kandalaksha Bay, White Sea, Russia). Coelomic epithelium was obtained by cutting out epithelium from the aboral side of a starfish, as described in [31]. The tissue from the arm tip was obtained by making a ~5 mm cut in the area of the eye spot. Circulatory coelomocytes were collected by cutting off the arm tip and collecting the coelomic fluid into a tube with anticoagulant solution [76]. The cells were pelleted by centrifugation at 120× *g* for 10 min in a bucket rotor. All tissues were snap-frozen in liquid nitrogen and stored at −80 °C for further usage.

### 4.3. Mice Immunization

Anti-Lystar5 serum was obtained from two 10-week BALB/c male mice immunized intraperitoneally by 20 µg of recombinant Lystar5 dissolved in 100 µL of complete Freund’s adjuvant (FCA, BD Biosciences, Franklin Lakes, NJ, USA). After 7 days, mice were also immunized by 20 µg of recombinant Lystar5. After an additional 7 days, mice were euthanized in a CO_2_ chamber (Acrylmedic, Romashkovo, Russia), decapitated, and anti-Lystar5 serum was obtained from the blood clotted in RT for 30 min. All animal care and experimental procedures were performed in accordance with the guidelines set forth by the European Communities Council Directive of 22 September 2010 (2010/63/EU), https://eur-lex.europa.eu/legal-content/en/TXT/?uri=CELEX%3A32010L0063, accessed on 24 March 2025) and were approved by the Ethical Committee of IBCH RAS for the control of the maintenance and use of animals (protocol 379/2024).

### 4.4. ELISA

To investigate the active site of Lystar5, we used ELISA. Membrane fractions of different *A. rubens* tissues from four different starfish individuals were isolated as described in [60]. After that, the membrane fraction was dissolved in sodium bicarbonate buffer (pH = 9.3) and immobilized on the 96-well ELISA plates (9018, Corning, Corning, MS, USA) for 3 h (RT) at a concentration of 2 µg of total protein per well. Then, plates were blocked by 5% BSA (Biosera, Cholet, France) in PBS for 2 h (RT), washed (ELISA wash buffer, Mybiosource, San Diego, CA, USA), incubated with different concentrations of Lystar5 (10^−5^–10^−10^ M) or with peptides mimicking Lystar5’ loops and head (10^−9^–2 × 10^−5^) for 2 h, washed, incubated with anti-Lystar5 serum (1:100), for 1 h, washed, incubated with donkey anti-mouse HRP-conjugated antibody (715-035-150, Jackson Immunoresearch, West Grove, CA, USA, 1:10,000) for 1 h, washed, and incubated with TMB-One ELISA Substrate (Mybiosource). The reaction was stopped by 200 mM HCl, and O.D. was measured using AMR-100 microplate reader (Allsheng, Hangzhou, China) at 450 nm. The O.D. from the wells incubated with PBS was subtracted from the O.D. of the wells incubated with recombinant Lystar5. The O.D. curves were fitted using the 4-parameter Hill equation (bottom constrained to 0) of GraphPad Prism 10.6.0 software (GraphPad Software, San Diego, CA, USA). The calibration curves showing the antibodies’ dynamic range are given in Figure S9. For calibration curves, the different total membrane fractions of *A. rubens* coelomic epithelium or coelomocytes (a), or the different total membrane fractions of HaCaT keratinocytes (b) were immobilized on the wells of ELISA plate, and bound proteins were detected using antibodies as described.

### 4.5. Immunoprecipitation

To confirm the interaction of nAChRs and integrins in HaCaT cells, the antibodies that captured α3-nAChR (rabbit, ABIN1867228, Antibodies online, Aachen, Germany, 1:200), α4-nAChR (rabbit, ABIN5013334, Antibodies online, 1:200), α7-nAChR (mouse, ABIN5611363, Antibodies Online, 1:200), and β2-nAChR (mouse, ABIN5611311, Antibodies Online, 1:200) were immobilized in the 96-well ELISA plates (9018, Corning) overnight at 4 °C (total volume 50 µL, 0.25 µg of antibody per well). Then, the wells were blocked with 5% BSA for 2 h and incubated with the membrane fraction of HaCaT cells (obtained as in [60]) for 2 h. After that, integrin subunits bound to nAChRs were detected with the following rabbit primary antibodies: α5 integrin (4705, Cell Signaling, Danvers, MA, USA, 1:10,000), αV integrin (4711, Cell Signaling, 1:10,000), β1 integrin (ABIN7127584, Antibodies Online, 1:10,000), and β4 integrin (14803, Cell Signaling, 1:10,000). The plates were incubated with the primary antibodies for 2 h and with secondary HRP-conjugated goat anti-rabbit antibodies (111-035-003, Jackson Immunoresearch, 1:10,000) for 1 h. Then, the plates were washed and incubated with TMB-One ELISA Substrate (Mybiosource). The reaction was stopped by 200 mM HCl, and the O.D. was measured using an AMR-100 microplate reader (Allsheng) at 450 nm. Please note that for α3 and α4 nAChR subunits, we used rabbit antibodies for both capture and detection. To obtain the specific signal from integrin, the signal from empty wells without the addition of the membrane fraction was subtracted from the signal from the wells incubated with the membrane fraction of HaCaT cells.

### 4.6. Cell Cultivation

Human HaCaT keratinocytes (ATCC, Manassas, VA, USA) were grown (37 °C, 8% CO_2_) in a DME medium with phenol red (PanEco, Moscow, Russia), 10% fetal calf serum (Thermo Fisher Scientific, Waltham, CA, USA), and 2 mM L-glutamine (PanEco). Human peripheral blood monocytes were isolated from human donors’ blood (informed written consent was obtained from the donors) with Lymphosep media (MP Biomedicals, Irvine, CA, USA) and cultivated in RPMI-1640 medium with phenol red (PanEco, Russia), 10% fetal calf serum (Thermo Fisher Scientific), and 2 mM L-glutamine (PanEco). All cells were routinely tested for the absence of mycoplasma contamination (Mycoreport kit, Evrogen, Moscow, Russia).

### 4.7. Wound-Healing (Scratch) Assay

The in vitro wound healing (scratch) assay was performed as described in [35]. In brief, HaCaT cells were seeded in 96-well cell culture plates (5 × 10^4^ cells/well) and grown for 24 h. Then, the media from the wells was changed to serum-free media to minimize cell proliferation. After another 24 h of cultivation, the wells were scratched with a sterile 10 μL pipette tip. Then, the cells were washed and treated with 1 µM of Lystar5 or 10 µM of its mimetics (from 10 mM mQ stock solution) for 24 h. The longer incubation periods resulted in cells’ overgrowth, detachment and death. Pictures were analyzed after 0 and 24 h at 20× magnification at CloneSelect Imager (Molecular Devices, San Jose, CA, USA). The center of the plate was marked as a central reference point to ensure that the same area was recorded during the time course. Digital images were taken, and the scratch area was quantified using ImageJ 1.54f software (NIH, Bethesda, MD, USA) and MS Excel 365 (Microsoft, Redmond, WA, USA) software by measuring the % of the scratch surface occupied by migrating cells.

### 4.8. Transwell Cell Migration Assay

The transwell assay, in which cells migrated through 8 µm pores of polystyrene membrane, was used to study individual cell migration. Cells (HaCaT or PBMCs) were seeded in migration chambers in 24-well plates (SPL Lifesciences, Pocheon, Korea; 2 × 10^5^ cells/well), immediately treated with 1 µM of Lystar5 or 10 µM of its mimetics (from 10 mM water stock solution), and incubated for 24 h or 72 h without media change (the incubation time was chosen so that the cells did not die at the end of the experiment). Then, the cells migrating through the pores were photographed (100× magnification, CloneSelect Imager, Molecular Devices). The number of migrated cells was quantified using the find maxima option of the ImageJ 1.54f software (NIH).

### 4.9. Affinity Extraction and Western Blotting

Lystar5 or its loops I or II (1 mg/mL) were coupled with NHS Flexibind magnetic beads (LSKMAGN01, Millipore, Burlington, NJ, USA) according to the manufacturer’s instructions and blocked for 4 h with 500 mM of ethanolamine. The beads blocked by 500 mM ethanolamine without any coupled protein were used as a negative control (empty beads, Figures S2–S5). The beads conjugated with BSA (1 mg/mL) were used as a specificity control (Figure S6). The membrane fraction of HaCaT cells or PBMCs (5 × 10^7^ cells per sample) was isolated as described in [60] and pre-cleared by incubation with empty magnetic beads for 1 h. Then, the membrane fraction was incubated with the Lystar5/mimetics-coupled, empty, or BSA-conjugated beads for 1 h in TBS. After that, unspecific bound proteins were sequentially washed out from the beads with TBS, TBS + 1 M NaCl + 0.5% Triton X-100, and TBS + 0.5% Triton X-100. The specifically bound proteins were eluted by heating (95 °C, 10 min) in reducing PAGE buffer. Western blotting was performed as in [35]. The following targets were detected: α3-nAChR (rabbit, ABIN1867228, Antibodies online, 1:1000), α4-nAChR (rabbit, ABIN5013334, Antibodies online, 1:1000), α7-nAChR (mouse, ABIN5611363, Antibodies Online, 1:1000), β2-nAChR (mouse, ABIN5611311, Antibodies Online, 1:1000), α5 integrin (rabbit, 4705, Cell Signaling, 1:1000), αV integrin (rabbit, 4711, Cell Signaling, 1:1000), β1 integrin (rabbit, ABIN7127584, Antibodies Online, 1:1000), and β4 integrin (rabbit, 14803, Cell Signaling, 1:1000). The secondary antibodies were HRP-conjugated goat anti-rabbit (111-035-003, Jackson Immunoresearch, 1:5000) or goat anti-mouse (FNSA-0003, Finetest, Wuhan, China, 1:5000). The HRP signal was detected by the ECL substrate (Bio-Rad, Hercules, CA, USA) using the ImageQuant LAS 500 imaging system (GE Healthcare, Chicago, IL, USA). The ECL signal was visualized in the chemiluminescent channel, and the protein marker was detected in the optical channel of the imaging system.

### 4.10. Flow Cytometry

To determine the effect of Lystar5/mimetics on the expression of migration-related surface receptors and adhesion molecules, HaCaT keratinocytes or PBMCs (2 × 10^5^ cells in a 24-well culture plate) were treated with 1 µM of Lystar5 or 10 µM of its mimetics (from 10 mM mQ stock solution) and incubated for 24 h or 72 h without media change, detached by the Versene solution (for adhesive HaCaT cells), fixed in 4% paraformaldehyde, and incubated for 1 h with the rabbit primary antibodies for E-cadherin (3195, Cell Signaling, 1:2000), N-cadherin (13116, Cell Signaling, 1:2000), α5 integrin (4705, Cell Signaling, 1:2000), and αV integrin (4711, Cell Signaling, 1:2000), EGFR (FNab02667, Finetest, 1:2000) or with mouse primary antibodies to VEGFR2 (sc-6251, Santa Cruz, Dallas, TX, USA, 1:2000), ICAM-1 (sc-71292, Santa Cruz, 1:2000), VCAM-1 (sc-20070, Santa Cruz, 1:2000). The cells were then washed with PBS and incubated with the alpaca anti-rabbit Alexa-488-conjugated antibodies (611-545-215, Jackson Immunoresearch, 1:1000), or with goat anti-mouse FITC-conjugated antibodies (FNSA-0030, Finetest, 1:1000).

Cells stained only with the secondary antibodies were used as a negative control. All cells were analyzed by the Attune NxT flow cytometer (Life Technologies, Waltham, CA, USA) using the Attune NxT 2.3. Software (Life Technologies). The gating strategy is shown in Figure S10.

### 4.11. Analysis of Inflammatory Cytokines Secretion by HaCaT Cells and PBMCs

To study the effect of Lystar5/mimetics on the secretion of cytokines and intercellular adhesion molecules involved in cell adhesion by the HaCaT cells and PBMCs, the 6-plex Adhesion immunoassay kit (BMS812FF, Bender Medsystems, Vienna, Austria) was used. Media (25 μL) were collected from the cells treated for 24 h or 72 h with 1 µM of Lystar5 or 10 µM of its mimetics (from 10 mM water stock solution), immunoassayed according to the manufacturer’s protocol, and analyzed using the Attune NxT flow cytometer (Life Technologies) and the Attune NxT Software 2.3. (Life Technologies). To determine the concentrations of substances, a calibration curve (4-parameter non-linear regression) was built using GraphPad Prism 10.6.0. software (Figure S11). The gating strategy for the 6-plex adhesion immunoassay kit is shown in Figure S12.

### 4.12. Statistical Analysis

Data are presented as the mean ± SEM. The number of samples (biological replicates, n) and the number of statistical tests are indicated in the figure legends. The outliers were removed by the ROUT method (Q  =  10%). Before comparisons, the data were tested for normality (Shapiro–Wilk test, at *p* = 0.05). Differences in the data were considered statistically significant at *p* < 0.05. Analysis was performed using GraphPad Prism 10.6.0 software (GraphPad).

## 5. Conclusions

Here, we demonstrated for the first time that Lystar5 protein from *A. rubens* stimulates the migration and invasion of human skin keratinocytes and peripheral blood monocytes. The pro-migratory effect in the keratinocytes is at least partially promoted by expression upregulation of E-cadherin, α5, and αV integrins, and EGFR, and by upregulation of E-selectin secretion. The active site of Lystar5 is formed by loops I and II, and the peptides mimicking these loops resemble the pro-migratory activity of the starfish protein. Moreover, we showed that in skin keratinocytes and PBMCs, integrins form complexes with nAChRs, the previously suggested target of Lystar5. Targeting of the components of this signaling network by Lystar5 or its mimetics may be a prospective strategy for wound healing.

## Figures and Tables

**Table 1:** The fit parameters describing the dose–response curves for the binding of Lystar5 and the peptides mimicking loops I and II to the immobilized membrane fraction of the coelomic cells. Curves were fitted with the Hill equation (the bottom was constrained to 0) using GraphPad Prism 10.6.0 software. Data are presented as a parameter ± SEM (*n* = 4). **** (*p* < 0.0001) indicates the significant difference from Lystar5 parameters according to F-test.

Tissue	Parameter	Lystar5	Loop I	Loop II
Coelomocytes	A_1_	2.2 ± 0.07	2.31 ± 0.09	2.38 ± 0.17
	EC_50_, nM	67.13 ± 10.83	553.06 ± 103.02 ****	883.16 ± 258.51 ****
	nH	0.99 ± 0.14	0.98 ± 0.15	0.84 ± 0.16
Coelomic epithelium	A_1_	2.3 ± 0.09	2.06 ± 0.07	2.54 ± 0.13
	EC_50_, nM	42.05 ± 9.94	405.53 ± 68.37 ****	817.16 ± 181.89 ****
	nH	0.64 ± 0.07	1.09 ± 0.15	0.89 ± 0.15

## Data Availability

Data is contained within the article or supplementary material.

## Supplementary Materials

The following supporting information can be downloaded at https://www.mdpi.com/article/10.3390/md24010003/s1, Figure S1. Multiple sequence alignment of integrin α8-like protein from starfish *Asterias rubens* (upper line, GenBank accession code XP_033639394.1) and human α5 integrin (middle line, UniProt accession code P08648) and αV integrin (bottom line, UniProt accession code P06756); Figure S2. Whole Western-blotting membranes for analysis of nAChR subunits extracted by Lystar5, its loops I and II from the membrane fraction of HaCaT keratinocytes (*n* = 3 independent portions of cells); Figure S3. Whole Western-blotting membranes for analysis of nAChR subunits extracted by Lystar5, its loops I and II from the membrane fraction of PBMCs (*n* = 3 independent portions of cells); Figure S4. Whole Western-blotting membrane for analysis of integrin subunits extracted by Lystar5, its loops I and II from the membrane fraction of HaCaT keratinocytes (*n* = 3 independent portions of cells); Figure S5. Whole Western-blotting membrane for analysis of integrin subunits extracted by Lystar5, its loops I and II from the membrane fraction of PBMCs (*n* = 3 independent portions of cells); Figure S6. Whole Western-blotting membrane for analysis of specificity of affinity extraction in HaCaT (left) and PBMCs (right); Figure S7. Representative cell distribution histograms showing the expression of migration-related surface receptors and factors in HaCaT keratinocytes upon incubation during 24 h (a) and 72 h (b) and in PBMCs upon incubation during 24 h (c); Figure S8. Characterization of the peptides mimicking the Lystar5 loops and “head” regions; Figure S9. The calibration curves showing the dynamic range of serum used for detection of Lystar5/peptides (a) and antibodies used for ELISA with HaCaT membrane fraction (b); Figure S10. The gating strategy for flow cytometry experiments; Figure S11. The regression curves used for interpolation of cytokines secretion; Figure S12. The gating strategy for 6-plex adhesion flow cytometry kit.

## Abbreviations

The following abbreviations are used in this manuscript:

Ach	Acetylcholine.
BLASTP	Basic local alignment search tool for proteins.
EC_50_	Half-maximal effective concentration.
ECM	Extracellular matrix.
EGFR	Epidermal receptor growth factor.
ELISA	Enzyme-linked immunosorbent assay.
HPLC	High-performance liquid chromatography.
ICAM-1	Intercellular adhesion molecule 1.
IP3K	Inositol 1,4,5-trisphosphate 3-kinase.
Ly6/uPAR	Lymphocyte antigen-6/urokinase-type plasminogen activator receptor.
MALDI-MS	Matrix-assisted laser desorption/ionization mass spectrometry.
nAChR	Nicotinic acetylcholine receptor.
NMR	Nuclear magnetic resonance spectroscopy.
O.D.	Optical density.
PBMC	Peripheral blood mononuclear cell.
SEM	Standard error of the mean.
sICAM-1	Secreted intercellular adhesion molecule 1.
sICAM-3	Secreted intercellular adhesion molecule 3.
SDS-PAGE	Sodium dodecyl sulfate polyacrylamide gel electrophoresis.
SLURP-1	Secreted Ly-6/uPAR-related protein 1.
SLURP-2	Secreted Ly-6/uPAR-related protein 2.
sPECAM-1	Secreted platelet endothelial cell adhesion molecule 1.
sVCAM-1	Secreted vascular cell adhesion molecule 1.
VCAM-1	Vascular cell adhesion molecule 1.
VEGFR2	Vascular endothelial growth receptor 2.
