# Supplementary material for: Ly6/uPAR Protein from Asterias rubens Starfish Stimulates Migration and Invasion of Human Epithelial and Immune Cells
Source: Mar Drugs. 2025 Dec 19;24(1):3. doi: 10.3390/md24010003 (PMC12843386; doi:10.3390/md24010003)
Supplement: Supplementary file 1 [file marinedrugs-24-00003-s001.zip › marinedrugs-4008955-supplementary.pdf]

Supplementary information

Ly6/uPAR Protein from *Asterias rubens* Starfish Stimulates Migration and Invasion of Human Epithelial and Immune Cells

Ekaterina N. Lyukmanova [1,2,3,4,*], Tamara Y. Gornostaeva [2,3], Sergey V. Shabelnikov [5], Zakhar O. Shenkarev [2,3], Mikhail P. Kirpichnikov [1,4], Alexander S. Paramonov [2] and Maxim L. Bychkov [2]

[1] Faculty of Biology, Shenzhen MSU-BIT University, Shenzhen 518172, China; kirpichnikov@inbox.ru
[2] Shemyakin-Ovchinnikov Institute of Bioorganic Chemistry, Russian Academy of Sciences, Moscow 117997, Russia; gornostaevatamara@gmail.com (T.Y.G.); zakhar-shenkarev@yandex.ru (Z.O.S.)
[3] Moscow Center for Advanced Studies, Moscow 123592, Russia
[4] Interdisciplinary Scientific and Educational School of Moscow University «Molecular Technologies of the Living Systems and Synthetic Biology», Faculty of Biology, Lomonosov Moscow State University, Moscow 119234, Russia
[5] Institute of Cytology, Russian Academy of Sciences, Tikhoretsky Prospect 4, St. Petersburg 194064, Russia; buddasvami@gmail.com
* Correspondence: lyukmanova_ekaterina@smbu.edu.cn

***Asterias rubens* integrin α8 like protein (XP_033639394.1)**
***Homo sapiens* α5 integrin (P08648)**
***Homo sapiens* αV integrin (P06756)**

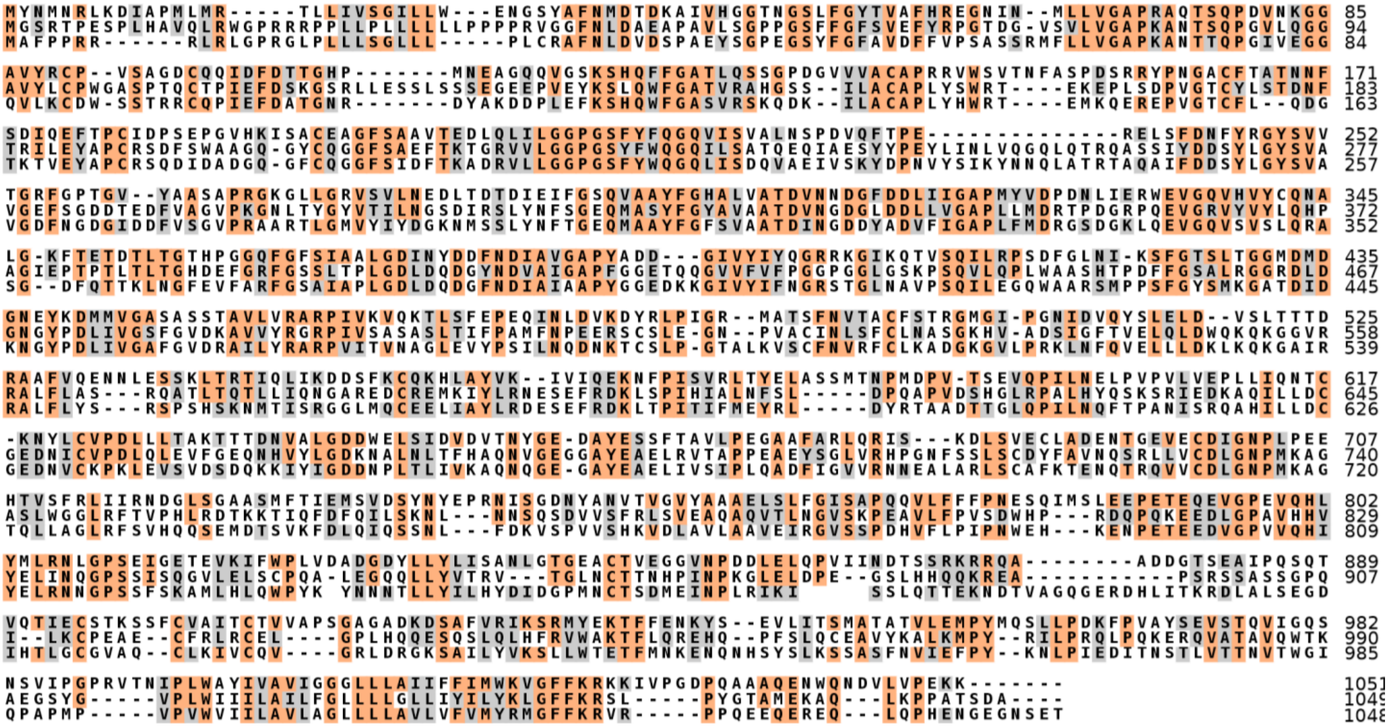

Figure S1. Multiple sequence alignment of integrin α8 like protein from starfish *Asterias rubens* (upper line, GenBank accession code XP_033639394.1) and human α5 integrin (middle line, UniProt accession code P08648) and αV integrin (bottom line, UniProt accession code P06756). Orange color denotes identical residues in *Asterias rubens* integrin α8 like protein and human integrins. Grey background marked the similar residues. Pair of residues was considered similar in the case of positive similarity score for the corresponding pair in the BLOSUM62 substitution matrix.

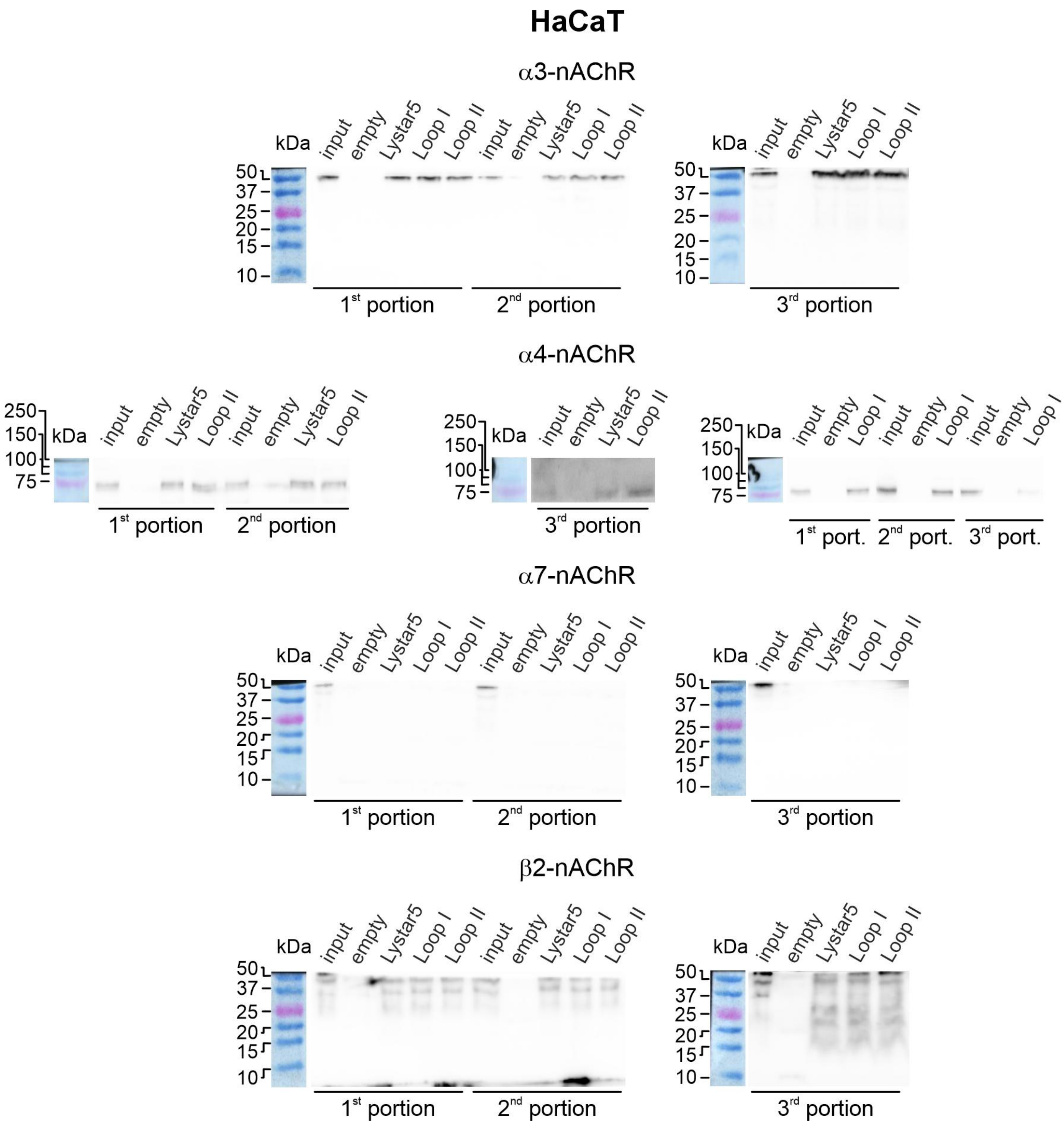

Figure S2. Whole Western-blotting membranes for analysis of nAChR subunits extracted by Lystar5, its loops I and II from the membrane fraction of HaCaT keratinocytes (n = 3 independent portions of cells).

PBMC

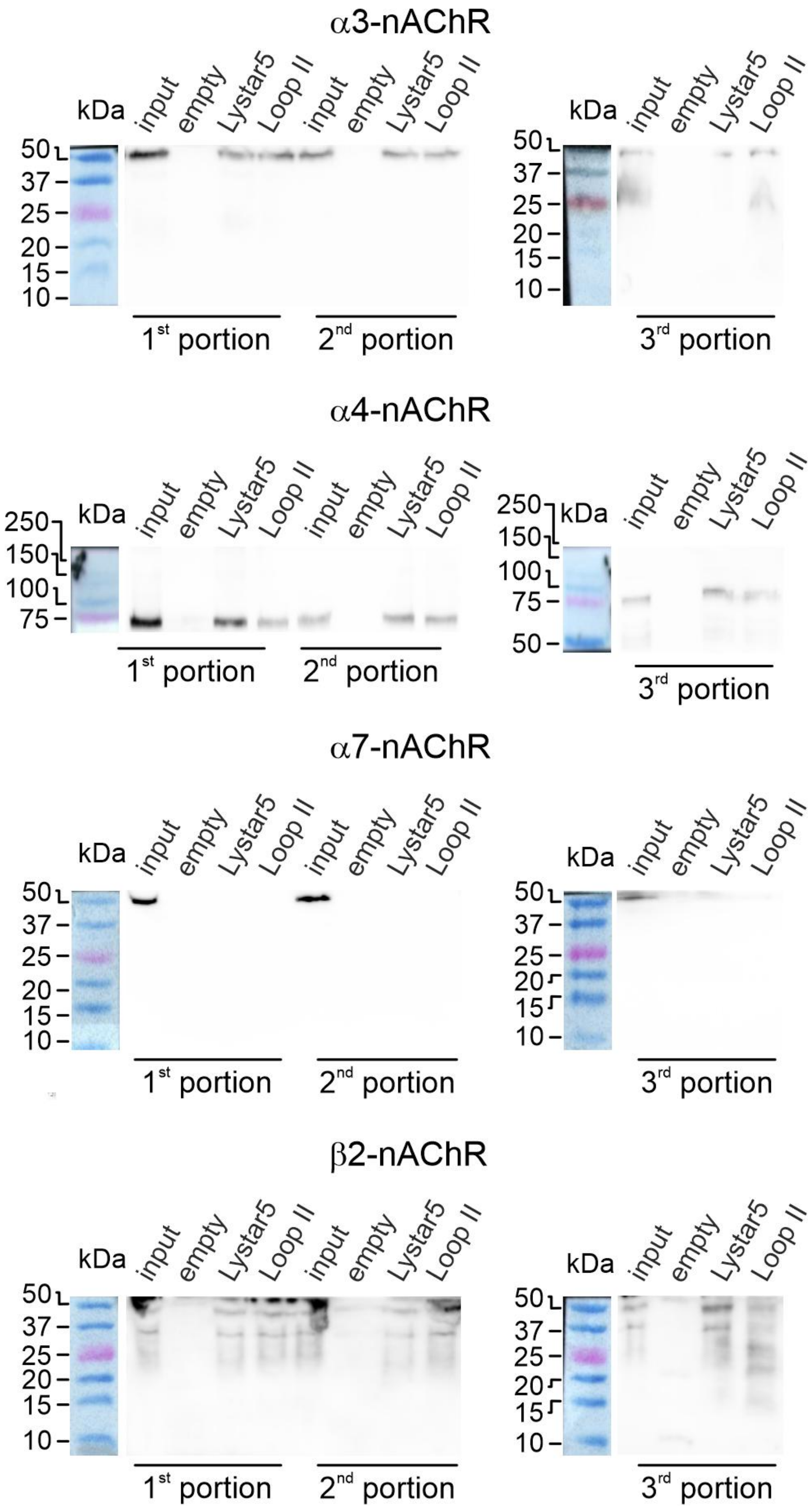

Figure S3. Whole Western-blotting membranes for analysis of nAChR subunits extracted by Lystar5, its loops I and II from the membrane fraction of PBMCs (n = 3 independent portions of cells).

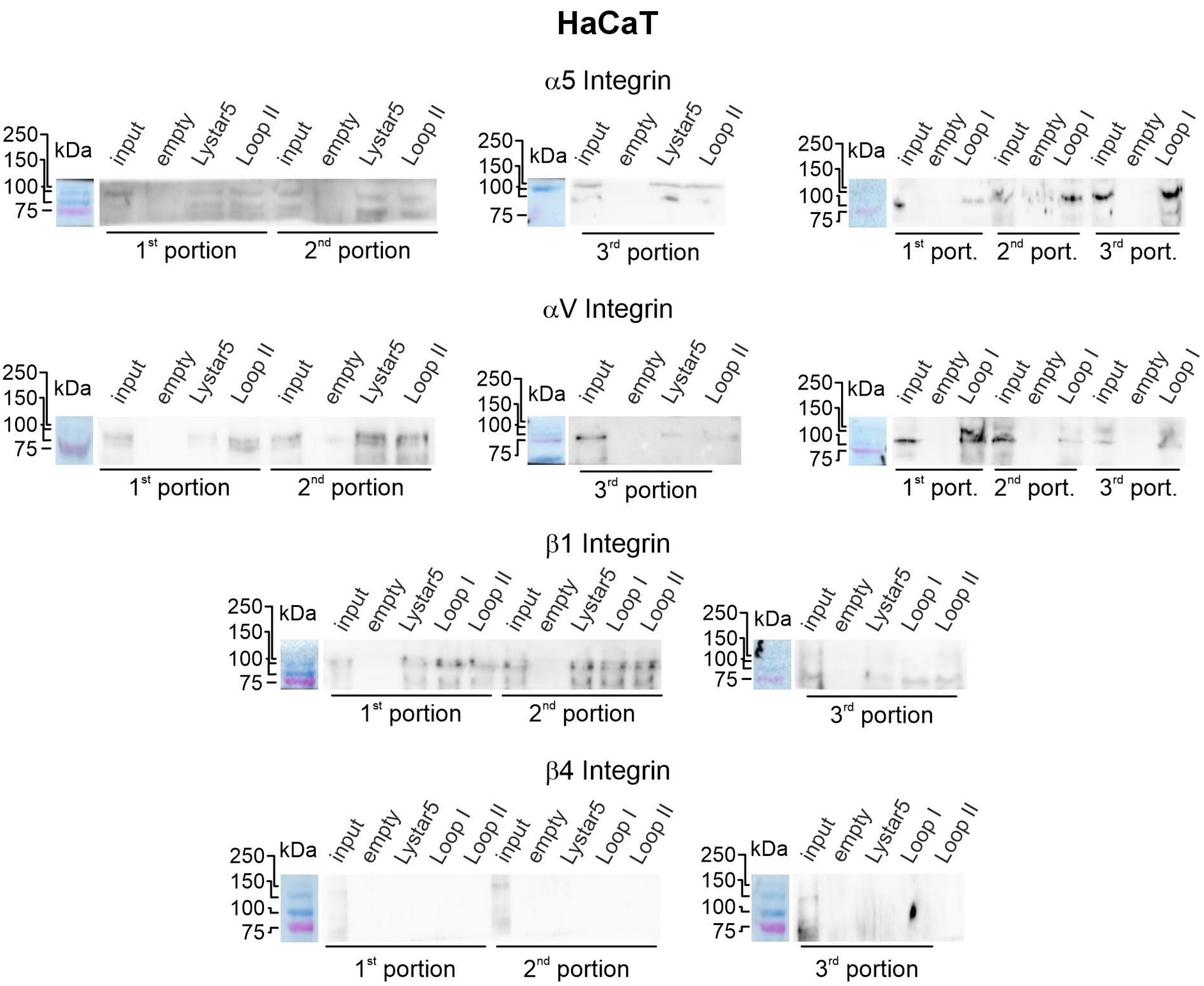

Figure S4. Whole Western-blotting membrane for analysis of integrin subunits extracted by Lystar5, its loops I and II from the membrane fraction of HaCaT keratinocytes (n = 3 independent portions of cells).

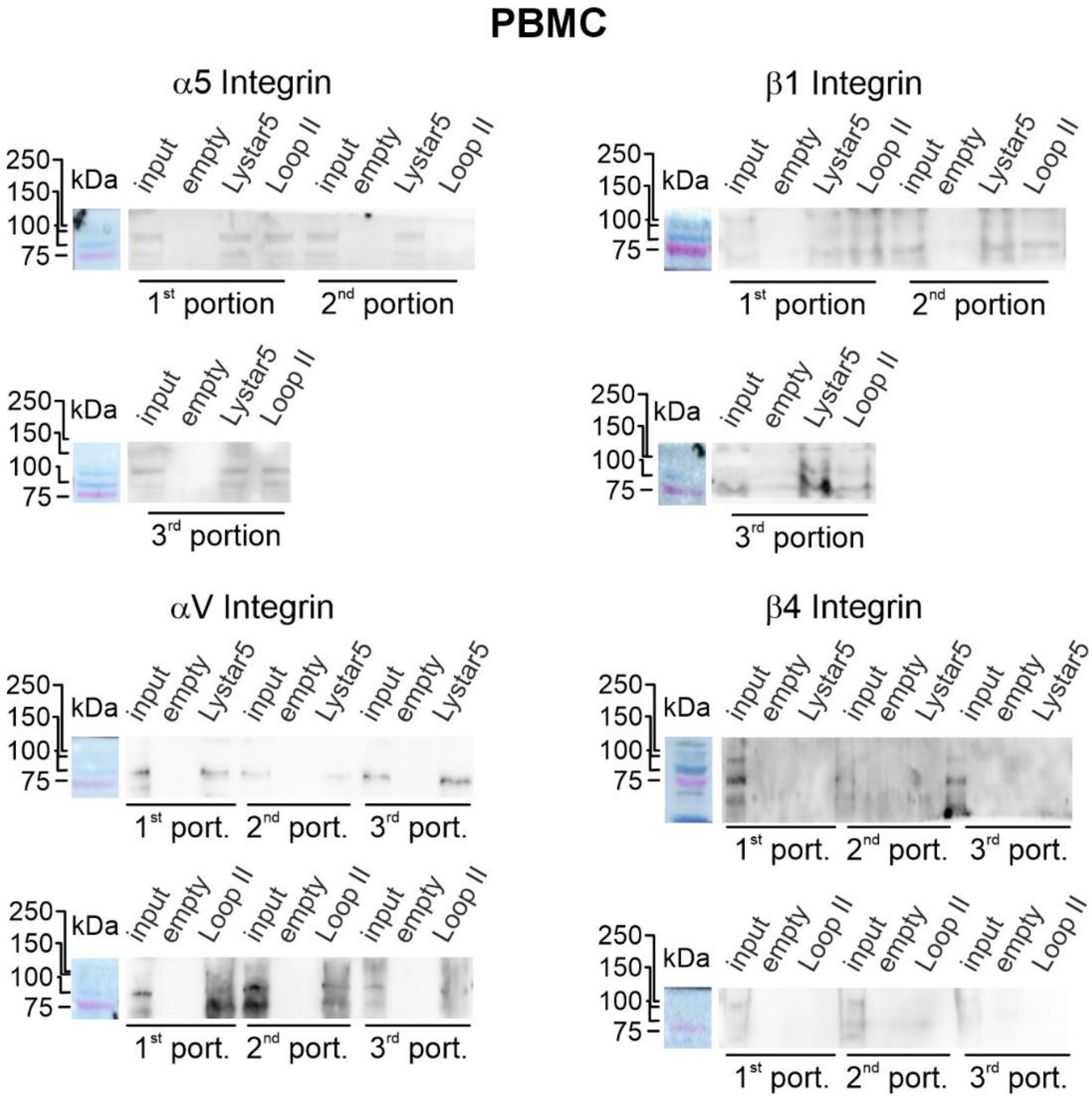

Figure S5. Whole Western-blotting membrane for analysis of integrin subunits extracted by Lystar5, its loops I and II from the membrane fraction of PBMCs (n = 3 independent portions of cells).

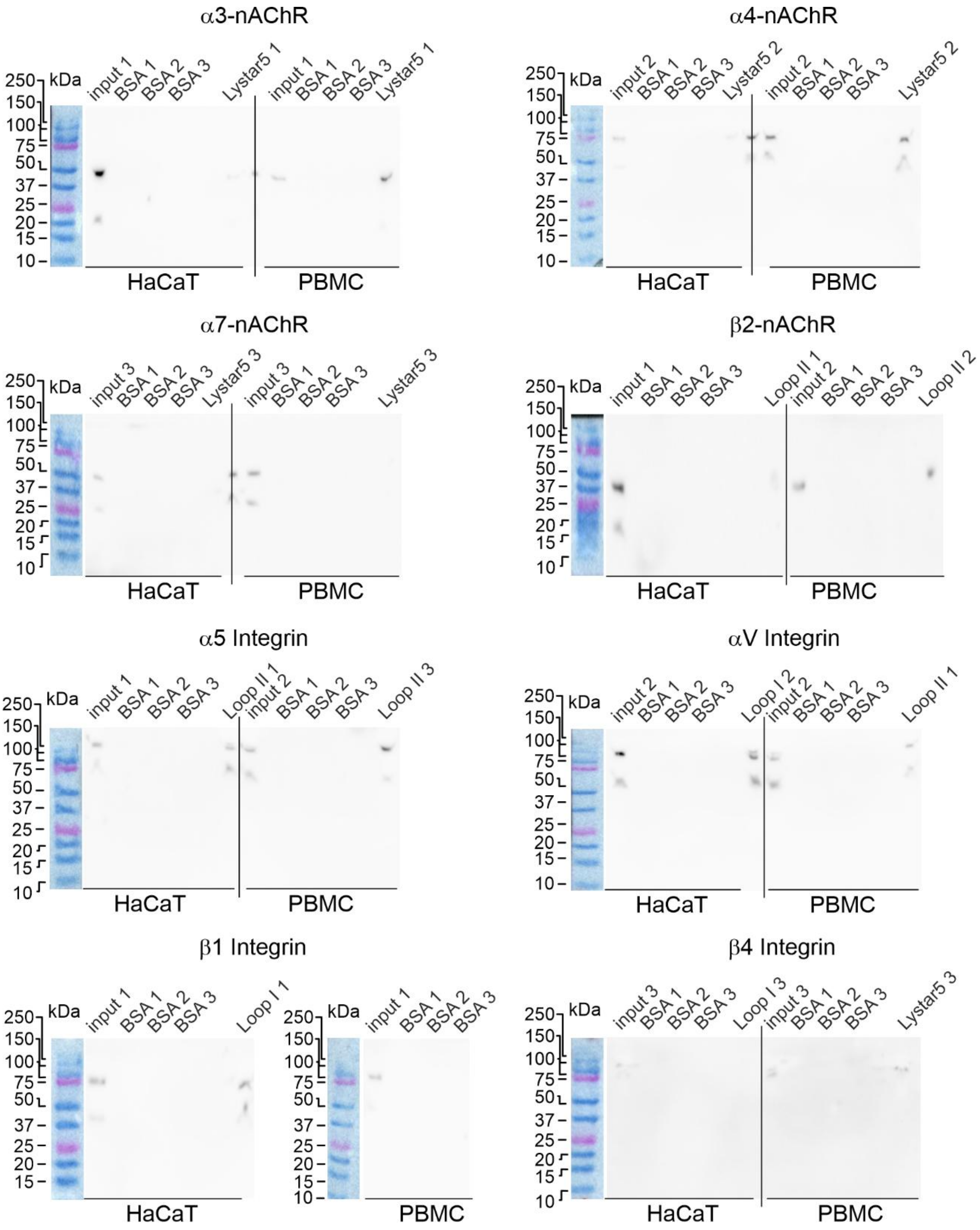

Figure S6. Whole Western-blotting membrane for analysis of specificity of affinity extraction in HaCaT and PBMCs. Some inputs and elutions from Lystar5/peptide-conjugated beads from membranes showed at Figures S2-S5 are also loaded. The vertical line designates extractions from HaCaT (left) and PBMCs (right).

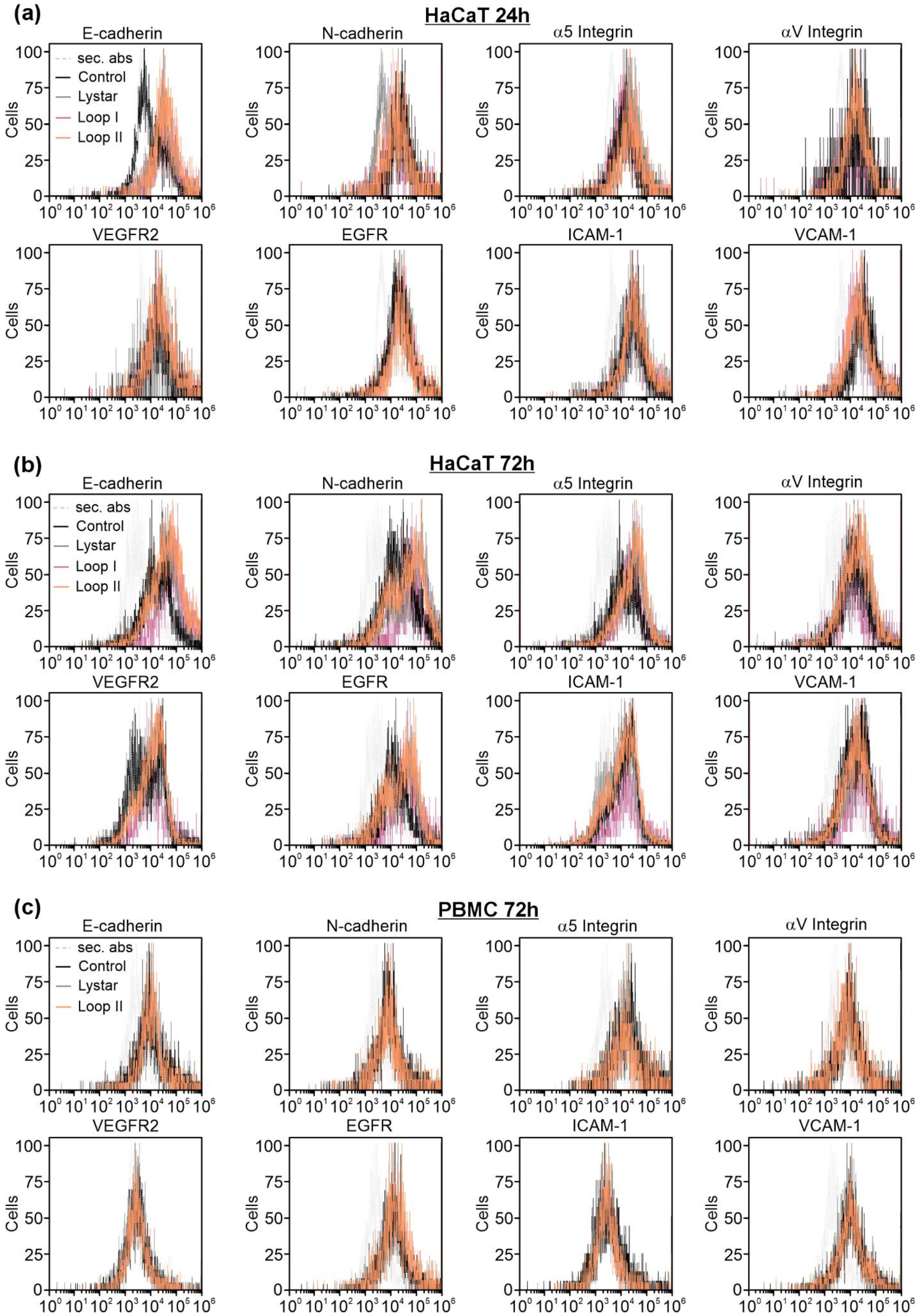

Figure S7. Representative cell distribution histograms, showing the expression of migration-related surface receptors and factors in HaCaT keratinocytes upon incubation during 24 h (a) and 72 h (b) and in PBMCs upon incubation during 24 h (c).

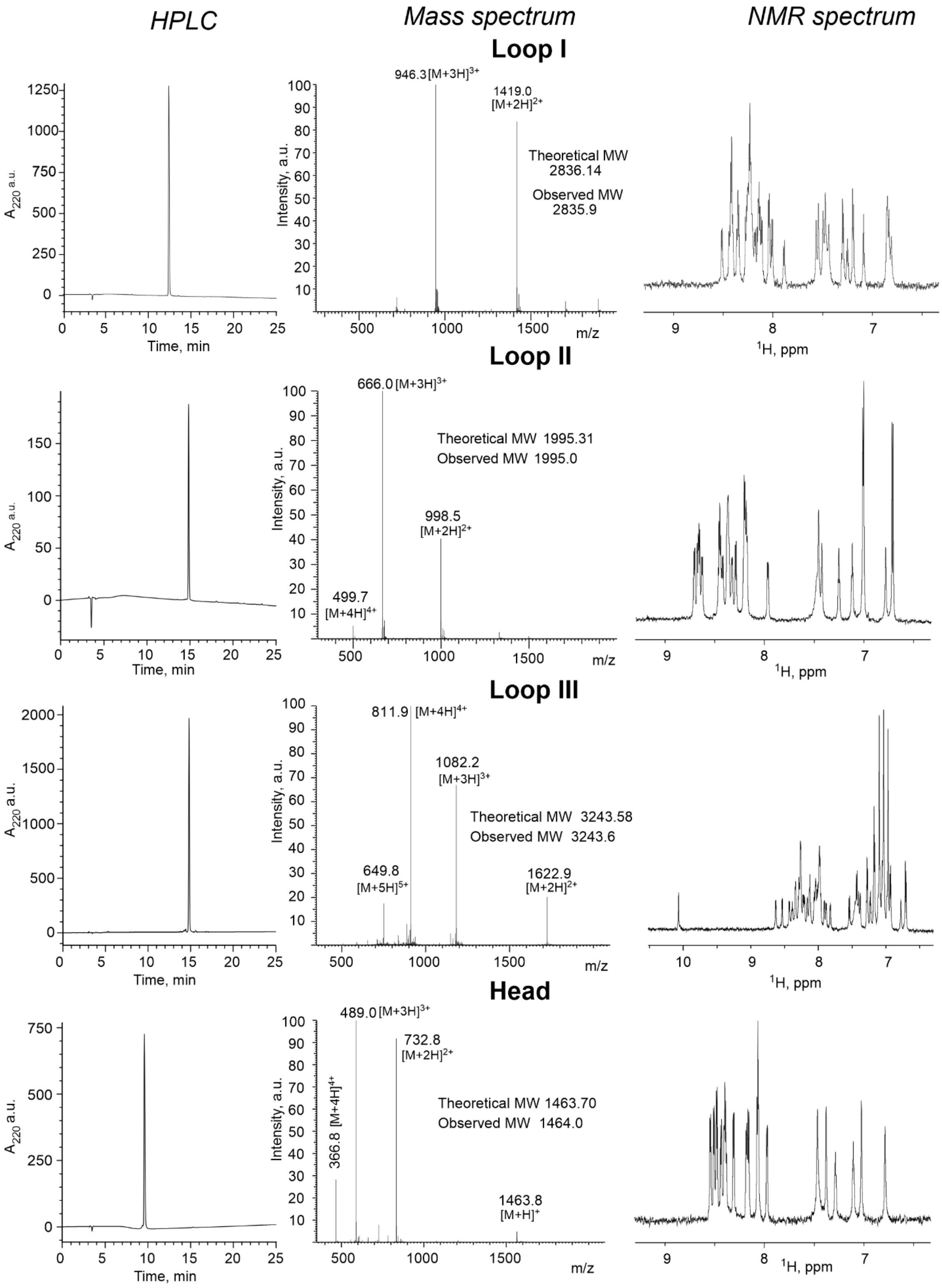

Figure S8. Characterization of the peptides mimicking the Lystar5 loop and "head" regions. **Left panel** – HPLC chromatogram (provided by vendor), 4.6 x 250 mm Inertsil ODS-3 column (GL Sciences, China), gradient 5-65% in 0.065% TFA at flow rate 1 ml/min). **Middle panel**: ESI mass spectrum (provided by vendor); **Right panel**: ^{1}H NMR spectrum (800 MHz, 30° C), the region of amide and aromatic protons is shown.

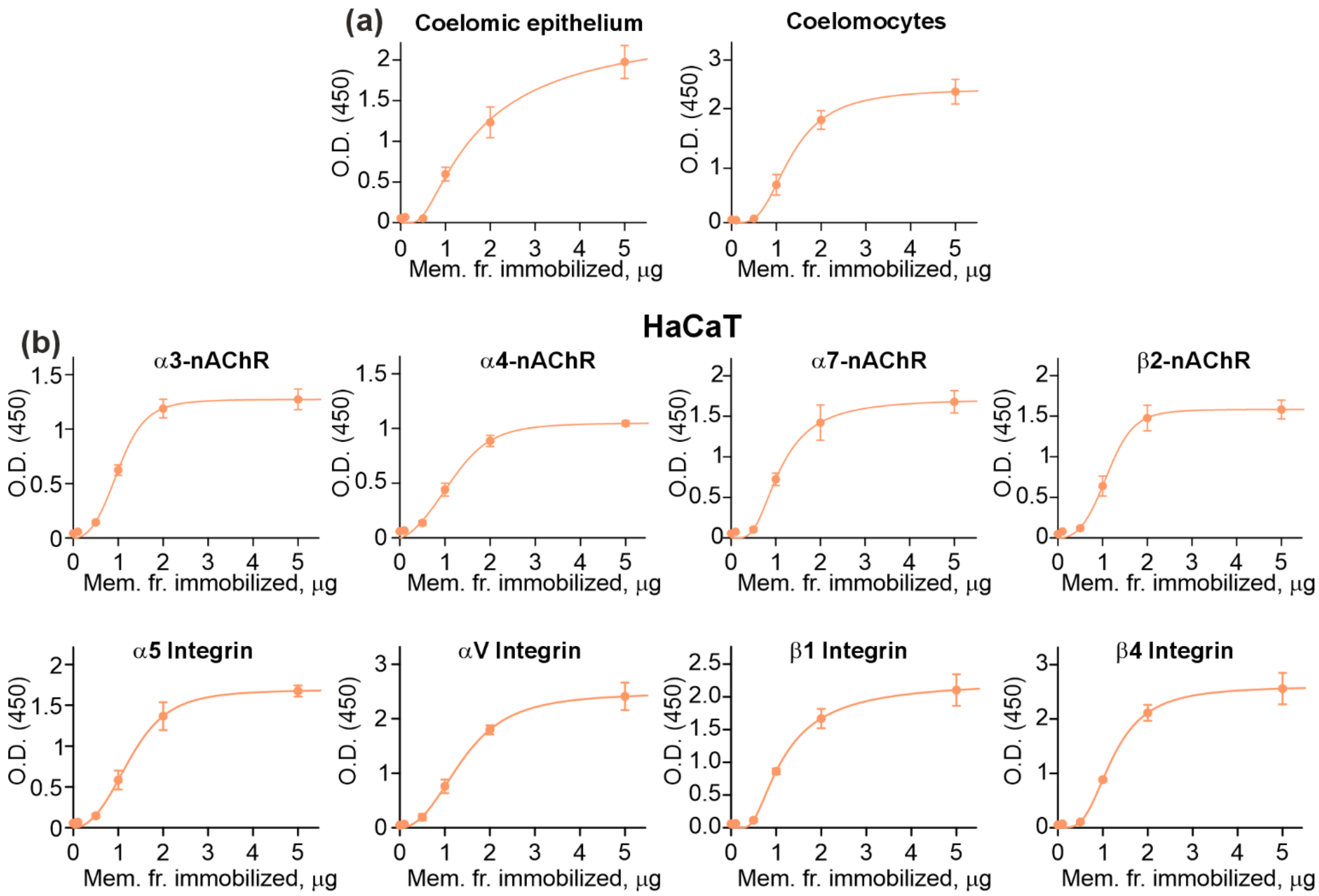

Figure S9. The calibration curves showing the dynamic range of serum used for detection of Lystar5/peptides (a) and antibodies used for ELISA with HaCaT membrane fraction (b).

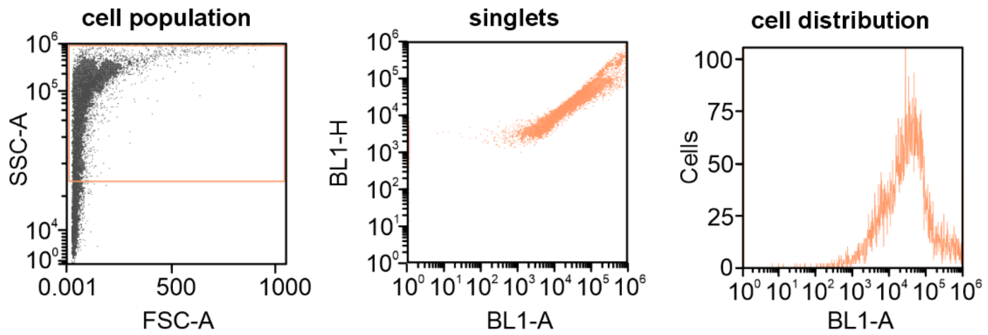

Figure S10. The gating strategy for flow cytometry experiments.

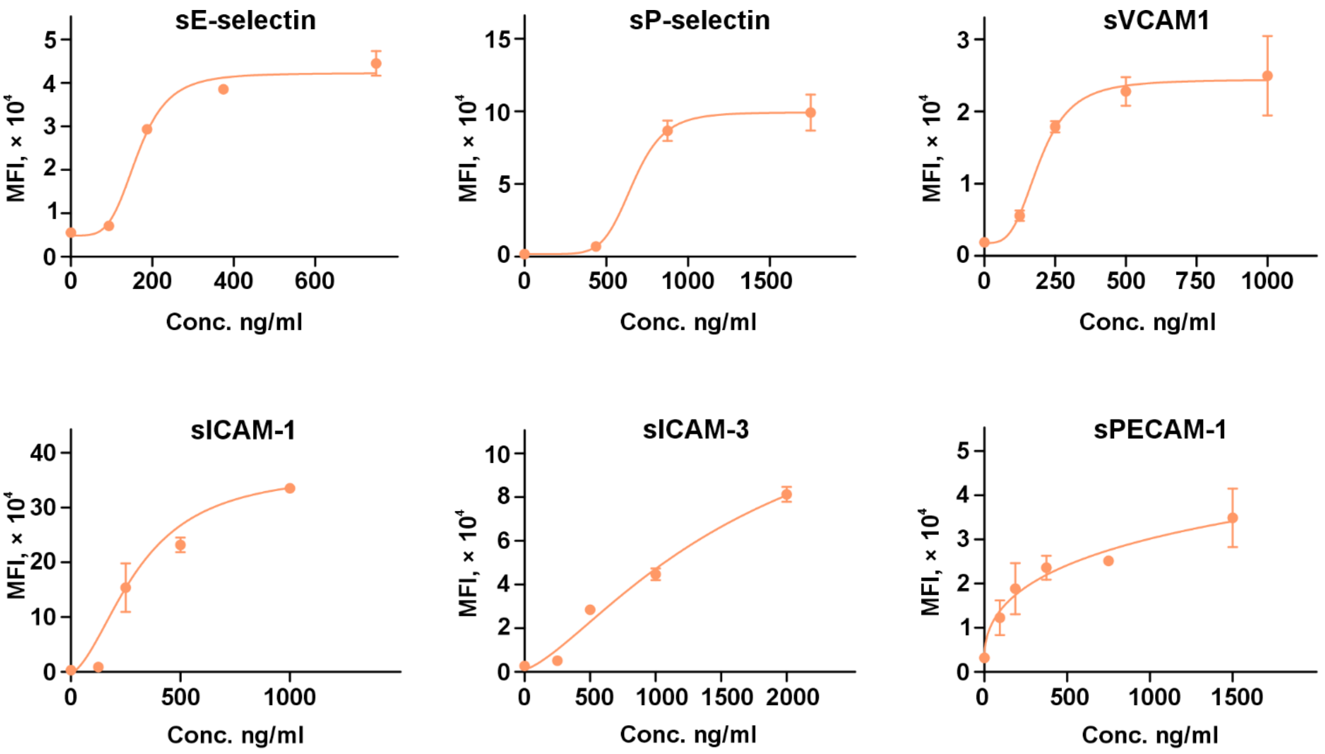

Figure S11. The regression curves used for interpolation of cytokines secretion.

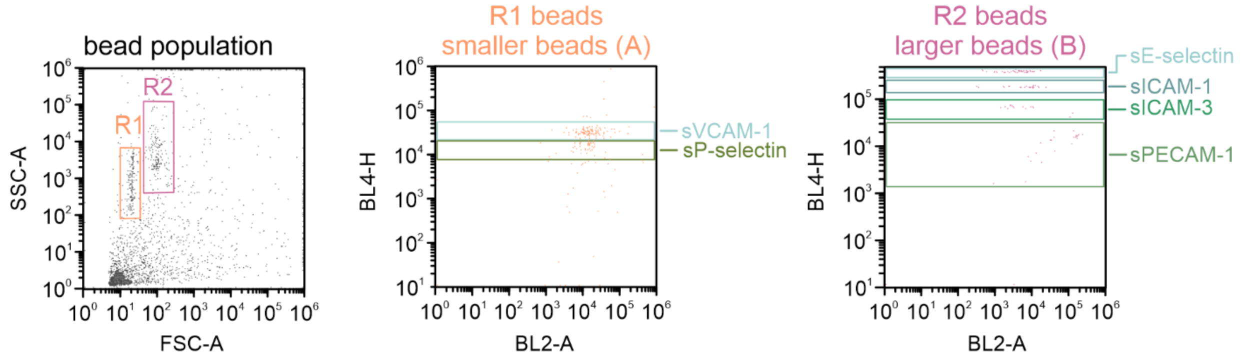

Figure S12. The gating strategy for 6-plex adhesion flow cytometry kit.

Disclaimer/Publisher's Note: The statements, opinions and data contained in all publications are solely those of the individual author(s) and contributor(s) and not of MDPI and/or the editor(s). MDPI and/or the editor(s) disclaim responsibility for any injury to people or property resulting from any ideas, methods, instructions or products referred to in the content.
